# A Review of Print Heads for Fused Filament Fabrication of Continuous Carbon Fiber-Reinforced Composites

**DOI:** 10.3390/mi15040432

**Published:** 2024-03-24

**Authors:** Heng Cai, Yuan Chen

**Affiliations:** 1 Shenzhen Key Laboratory of Intelligent Manufacturing for Continuous Carbon Fiber Reinforced Composites, Southern University of Science and Technology, Shenzhen 518055, China; caih@sustech.edu.cn; 2 School of System Design and Intelligent Manufacturing (SDIM), Southern University of Science and Technology, Shenzhen 518055, China

**Keywords:** print head, fused filament fabrication, continuous carbon fiber-reinforced composites, extrusion method, additive manufacturing

## Abstract

The print head is one of the most critical components in an additive manufacturing (AM) system. It can significantly affect the quality of printed parts. Recently, because continuous carbon fiber-reinforced composites can have excellent mechanical properties, a relevant AM technique, fused filament fabrication (FFF), has been attracting increasing attention. This has extended the requirements demanded of print heads. To this end, different FFF extrusion methods have been rapidly developed based on various methods of impregnating fibers into the matrix for the corresponding print heads. Generally, these extrusion methods are of three types: single extrusion, in situ extrusion, and dual extrusion. All these methods face substantial challenges, such as the nozzle clogging and damage to the continuous carbon fibers during extrusion. These common issues still need to be fully addressed. This study’s aim is to summarize and discuss the different extrusion methods and their FFF specific components in terms of their advantages and disadvantages for continuous carbon fiber-reinforced composites.

## 1. Introduction

**Figure 1:**
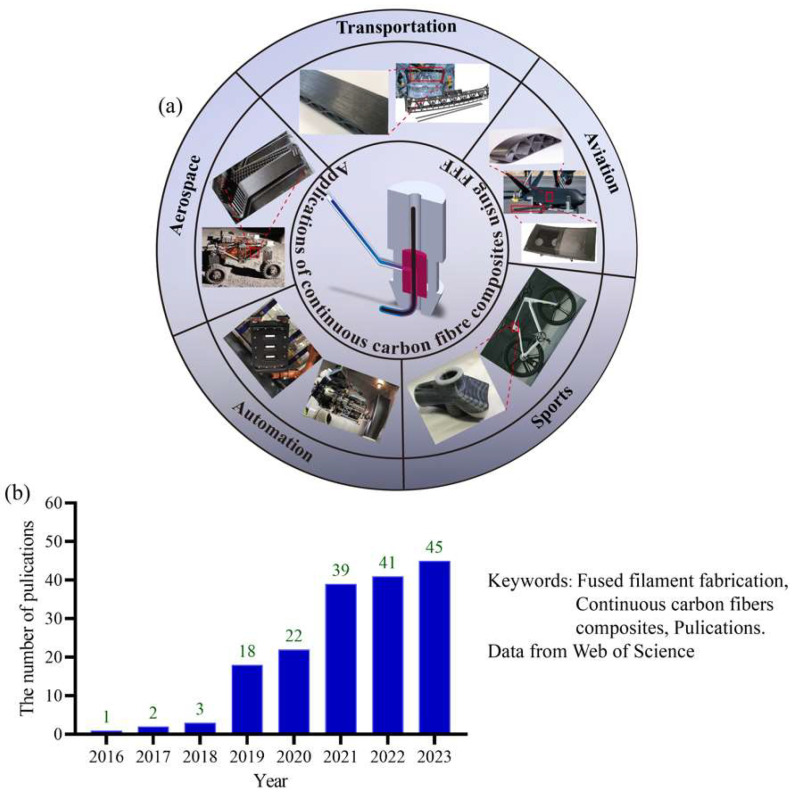
Investigation into continuous carbon fiber-reinforced composites using FFF: (**a**) engineering applications and (**b**) relevant publications based on Web of Science.

**Figure 2:**
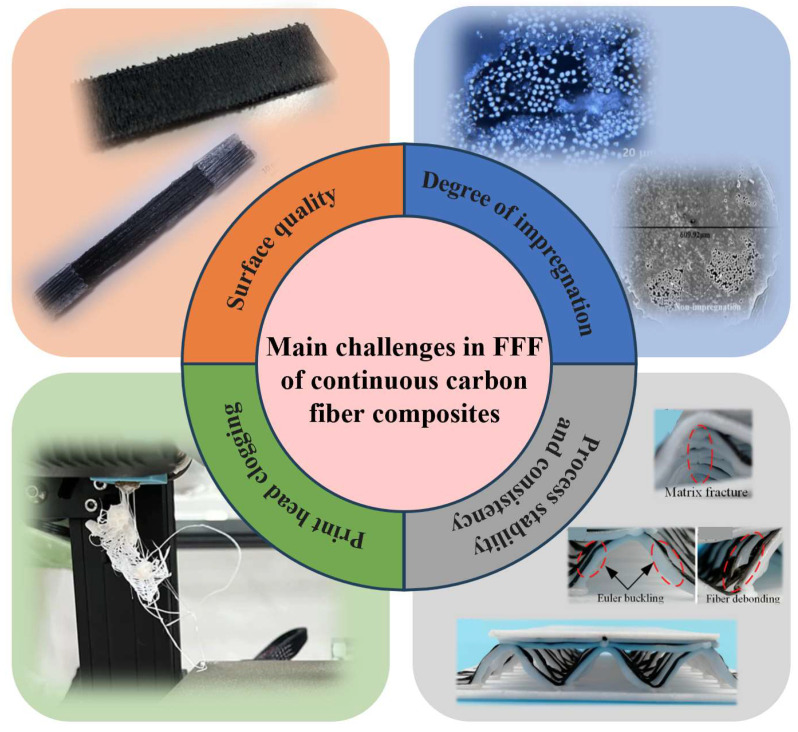
The main challenges in the fused filament fabrication of continuous carbon fiber-reinforced composites [9,10,11].

Historically, the complexity of manufacturing parts has been a major factor driving cost. Recently, advancements in additive technologies have led to a shift from rapid prototyping to the production of actual end-use parts, considerably widening the design possibilities [1]. However, with the materials currently utilized in additive manufacturing, it is difficult to fully exploit the technological potential of the process [2]. In comparison to polymers, short carbon fiber-reinforced composites exhibit enhanced mechanical properties, including improved stiffness, strength, and impact resistance, albeit with some anisotropic behaviors due to the random fiber orientation [3]. In contrast, continuous carbon fiber composites generally demonstrate superior mechanical properties relative to short fiber composites, especially concerning strength and stiffness along the fiber direction [4]. Continuous carbon fibers are introduced in the fused filament fabrication (FFF) process to unlock the limitation of mechanical properties, satisfying the operational conditions for aerospace and transportation [5,6], as shown in Figure 1a. By employing the freeform path planning technique of FFF technology, optimizing the printing directions for printing continuous fiber composites is crucial for achieving superior mechanical properties, reducing defects, enhancing efficiency, and customizing the functionality of the printed parts. By searching in the Web of Science using fuse filament manufacturing and continuous carbon fiber composites, investigation into continuous carbon fiber-reinforced composites in FFF is attracting increasing attention, as Figure 1b shows [7,8]. As shown in Figure 2, the main challenges in the fused filament fabrication of continuous carbon fiber-reinforced composites are attributed to factors such as print head clogging, the degree of impregnation [9,10], surface quality, process stability and consistency [11], etc. These issues predominantly depend on the coordination of the additive manufacturing equipment and process parameters, as well as the specific extrusion methods. However, the equipment suitable for manufacturing continuous carbon fiber-reinforced composites is currently still in development, with a strong emphasis on advancing the key print head technologies [12]. The structural design of the print head is crucial for ensuring the precise and consistent deposition of continuous carbon fiber-reinforced composites during the printing process [13].

The fabrication of continuous carbon fiber composites in the FFF process involves a series of intricate parameter settings closely associated with the structural design of the print head. In practice, the print parameters, such as the print temperature, the width and height of the print path, and the print speed, have a direct impact on the quality of molding and the mechanical performance of the printed continuous carbon fiber composite parts [14]. In other words, the molding accuracy and mechanical properties of the printed part are directly influenced by the operational parameters and internal structure of the print head.

It is well known that there is a considerable gap between the mechanical properties of continuous carbon fiber-reinforced composites manufactured through FFF technology and those produced using traditional processes [15,16]. This can be attributed to the low volume fraction of continuous carbon fibers, poor wettability, a damaged reinforcement phase, and pore defects at the adjacent printed filament [17]. To address these issues, different extrusion methods (single extrusion, in situ co-extrusion, and dual extrusion) are employed in the print head to optimize the printing process of such continuous carbon fiber composites [18]. 

In addition, the rheological behaviors of melting polymers inside the print head are susceptible to fluid–structure coupling and thermo-mechanical coupling [19]. To overcome these challenges, it is necessary to customize the design of components, such as the nozzle, chamber, heating block, guide pipe, and guide pulley, to ensure uniform temperature distribution, efficient infiltration, and smooth flow inside the print head. Through empirical experimental techniques, it is feasible to acquire suitable processing parameters for the print head that proficiently tackle challenges during manufacturing. 

In this article, the relevant research, design, and progress of print heads for the FFF processing of continuous carbon fiber composites are systematically reviewed. A variety of internal structural designs, the functionalities of components, and the current limitations of print heads are comprehensively discussed. Furthermore, a forward-looking perspective on the development of print heads is provided, with an aim to fabricate parts composed of continuous carbon fiber composites with superior mechanical performance.

## 2. Print Heads with Different Extrusion Methods

**Figure 3:**
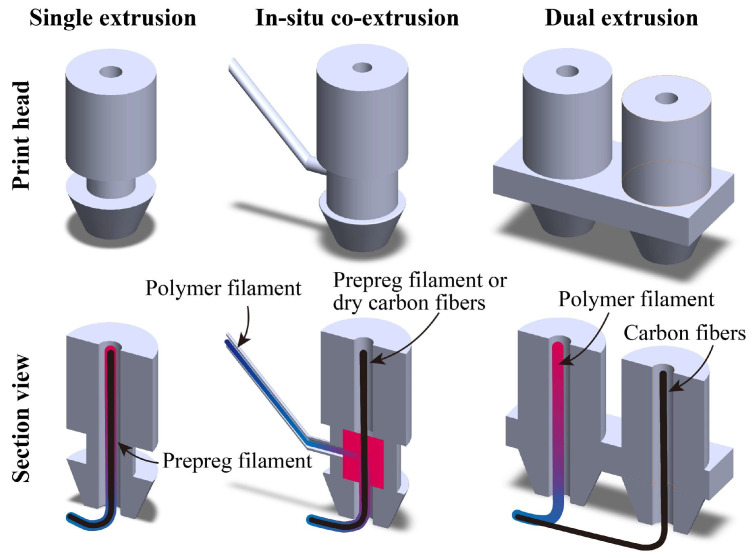
The different extrusion methods for print heads: single extrusion, in situ extrusion, and dual extrusion.

To improve the poor flowability of the matrix in pre-impregnated composites during the FFF process, various design proposals have been put forward for the print head—single extrusion, in situ co-extrusion, and dual extrusion, as shown in Figure 3. The cross-section of the print head in Figure 3 indicates that these designs primarily differ in the infiltration method utilized for the fiber and matrix. Whether the mixing process of continuous carbon fibers and melting resin occurs inside or outside the print head, the key premise is to ensure that the nozzles smoothly extrude the printing filaments. Here, the advantages and disadvantages of the three discussed extrusion methods (single extrusion, in situ extrusion, and dual extrusion) are described in Table 1. In the following sections, each extrusion method is explained further.

### 2.1. Single Extrusion

**Figure 4:**
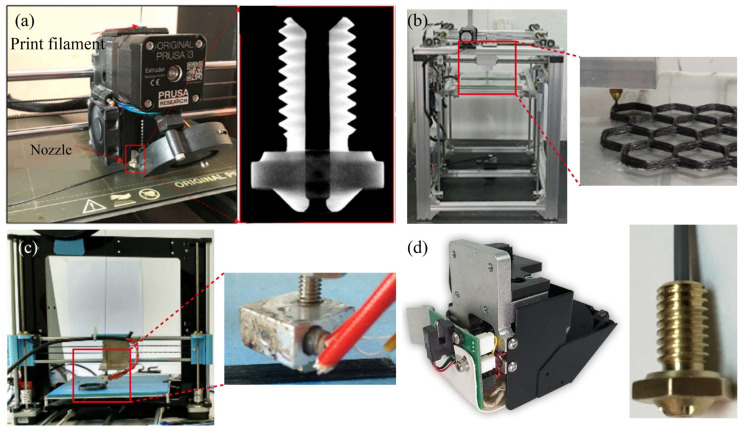
Self-developed print head for single extrusion method: (**a**) the modified Prusa i3 print head [22]; (**b**) the design of a small molten resin tank inside the print head [23]; (**c**) the choice of a larger nozzle [25]; (**d**) the print head and nozzle of a MarkTwo printer [28].

The structural design of the single extrusion print head is straightforward; it uses prepreg continuous carbon fiber composites as the printed filament. Zhuo et al. [20] reported the preparation process of prepreg filament, in which a commingled fiber tow was fed through a pultrusion die set at 250 °C, ensuring the melting of the thermoplastic fibers to impregnate the carbon fibers. Subsequently, the impregnated filaments were cooled and cured before being collected using an automated mechanism to consolidate the mixed fibers. Based on the filament preparation, Zhi et al. [21] developed a print head to print continuous carbon fiber-reinforced thermoplastic nylon polyamide-6 (PA6) polymers to improve the electromagnetic loss capacity of the printed sample. Generally, higher temperatures can result in nozzle clogging due to the accumulation of excess melted epoxy resin in the nozzle. Zhang et al. [22] successfully employed a Prusa i3 printer with a filleted brass nozzle to print carbon fiber-reinforced thermosetting epoxy resin at a temperature of 90 °C and a printing speed of 300 mm/min, as shown in Figure 4a. For a similar purpose, Ming et al. [23] designed a small molten resin tank inside the print head to impregnate the printed filament again, as seen in Figure 4b. Both thermosetting and thermoplastic resins are prone to nozzle clogging, a phenomenon associated with the rheological behavior of the molten resin within the print head. Excessive resin fluidity can have a negative impact on the molding quality of the composites, while inadequate fluidity can lead to the formation of more internal void defects within the composites.

A microscopic characterization test demonstrated that the design of the print head can lead to a significant reduction in the internal defects of the printed part [24]. In an effort to prevent clogging and filament breakage, Hu et al. [25] opted for a larger diameter nozzle of 1.5 mm during the printing process, as depicted in Figure 4c. To reduce friction between the printed filament and the tube wall in the print head, Sugiyama et al. [26] designed a polytetrafluoroethylene (PTFE) tube to pass through the extruder head from the entrance of the guide pipe to the end of the nozzle.

The input speed of the filament is less than the speed of the molten material flowing out of the nozzle, leading to potential manufacturing issues, such as improper feeding, fiber damage, and breakage. To this end, Akhoundi et al. [27] applied a single-extrusion print head to explore the motion of the fibers being influenced by the feed rate of the filament. Localized tension and potential damage of the continuous carbon fiber filament may be susceptible to localized friction and uneven feeding in the feed path, underscoring the importance of maintaining a consistent feed flow from the inlet to the outlet.

In commercially available additive manufacturing machines of continuous carbon fiber composites, the single-extrusion design is widely used in commercial printers, such as Markforged’s MarkTwo printer [28], as shown in Figure 4d. Chen et al. [29] found that the commercial filament used as “continuous fiber reinforcement” in the Markforged printer itself contains a fiber volume fraction of 34.5%. Therefore, the low-volume fraction of continuous carbon fibers used in fused filament fabrication is a critical constraint for high-performance composites. Adumitroaie et al. [30] demonstrated that the tensile elastic modulus and tensile strength of printed samples are 57.1 GPa and 528 MPa, respectively, at a fiber volume fraction of 35%. This print head, in conjunction with Markforged’s self-developed continuous carbon fiber-reinforced nylon composites, enables the creation of high-strength structural parts through the extrusion of a continuous carbon fiber bundle via a separate nozzle.

### 2.2. In Situ Co-Extrusion

**Figure 5:**
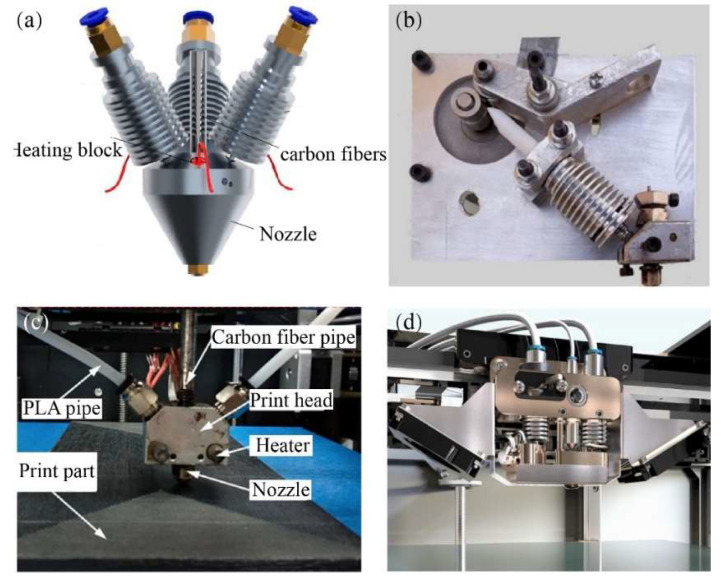
Self-developed print head of in situ co-extrusion method: (**a**) print head with three synchronously fed filaments [31]; (**b**) print head with feeder wheels [32]; (**c**) print head designed with consideration of chamber pressure [33]; (**d**) print head of Anisoprint printer [35].

The in situ co-extrusion technique is incorporated into the design of a single nozzle–double feed path, where continuous carbon fiber prepreg and matrix are simultaneously fed into the print head. Upon heating by the heat modulus, the matrix is soft and elastic, while the prepreg fibers turn sticky and lose some of their rigidity. The blend is extruded as a single composite, allowing it to bond with various kinds of thermoplastics and ensuring the high-quality formation of the printed filament. Kuschmitz et al. [31] developed a print head for processing continuous carbon fiber-reinforced polymers, where the fiber is guided through the molten flow of the polymer. In this case, three synchronously fed filaments are directed into the print head at an angle of 30°, as demonstrated in Figure 5a. E3D-V6 heat sinks are implemented to confine the filament melting process within the designated melting zone. The standard nozzle geometry has an inner diameter of 1.0 mm, with the inner edge rounded to prevent damage to the printed filaments. 

To preserve the quality of the feeding prepregs, Rarani et al. [32] incorporated feeder wheels to guide the prepreg filament into the print head, supporting control of the feed speed and ensuring that the proper stretching force is applied to the carbon fiber prepreg during the printing process, as depicted in Figure 5b. In addition, Wang et al. [33] observed that through precise control of the relationship between the prepreg diameter and the nozzle diameter, higher pressure can be achieved in the melt zone to effectively inhibit pores between adjacent printing filaments, as seen in Figure 5c.

In response to the low-volume fraction of continuous carbon fiber, Zhang et al. [34] adopted the co-extrusion scheme to achieve a continuous carbon fiber volume fraction above 20%. The Anisoprint commercial print head [35] utilizes a dual-feed print nozzle system, enabling the separate entry of the prepreg carbon fiber through corresponding feed paths, as shown in Figure 5d. This innovative technology allows for the use of thermosetting resin prepreg continuous fibers, coated with a layer of thermoplastic polymers as a binder. It can be seamlessly blended and extruded with various types of thermoplastic resin substrates. In comparison, the interfacial properties between the fibers and the matrix inside the prepreg are minimally affected. Adumitroaie et al. [36] demonstrated that the tensile elastic modulus and tensile strength of the printed sample are 60 GPa and 750 MPa, respectively, at a fiber volume fraction of 46%. The device is reported to enable the additive manufacturing of parts with a continuous fiber volume content of over 50%, and it is suitable for the 3D printing of high-performance aerospace-grade structural components [37].

Due to a lack of sufficient pressure and time during in situ impregnation, it has been difficult to satisfy the engineering requirements for the surface quality and mechanical properties of the printed parts. Kaczmarek et al. [38] used dry carbon fibers to complete in situ impregnation in the print head, but the quality of the printed part was poor. In general, an effective solution is provided by the in situ co-extrusion of the print head to overcome the limitations associated with the volume fraction of continuous carbon fibers in the additive manufacturing process. However, this imposes restrictions on the printing speed in order to guarantee molding quality and adequate blending within the print head, resulting in lower manufacturing efficiency [39].

### 2.3. Dual Extrusion

**Figure 6:**
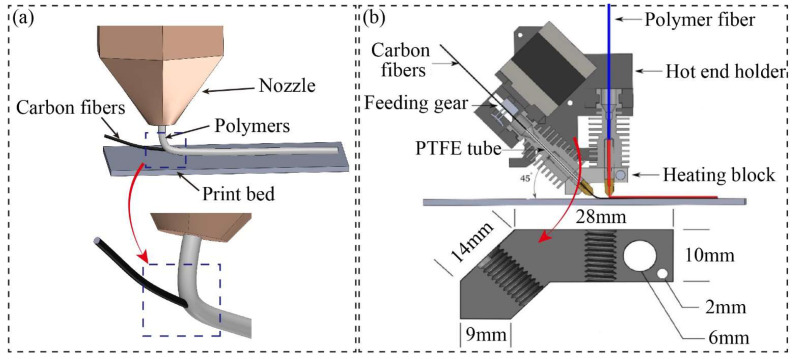
Schematic diagram of different dual-extrusion print heads: (**a**) the blending process between the carbon fibers and matrix outside of the print head; (**b**) the design of different angles with respect to the extrusion of polymers and carbon fibers [41].

For a print head with dual extrusion, the continuous carbon fiber prepreg is directed by a guide tube positioned close to the nozzle, as shown in Figure 6. Since the prepreg filaments are processed with less additional heating, the interface bonding between the fibers and the matrix can be preserved during the deposition of the primary prepreg filament. Further assurance of the bonding effect and printing efficiency between adjacent fused printing filaments is achieved through precise control of the printing temperature and speed. 

Saari et al. [40] introduced a new 3D printing technology where the fibers were aligned with the extrusion nozzle from the molten matrix, forming a composite print ribbon. This ensures coordination between the fiber supply speed and the extrusion nozzle speed, encapsulating the prepreg filaments directly into a flow of molten polymer, as seen in Figure 6a. The printed filament solidifies into a predetermined shape due to the toolpath. It hardens quickly, resulting in the fiber being trapped inside the extrudate and forming a ‘coaxial composite’. This process allows for simultaneous fiber encapsulation during polymer deposition, eliminating the need for any additional processing or post-processing time. 

With this aim, Olcun et al. [41] used an open-source printer (Prusa MK2 i3, Prusa Research, Prague, Czech Republic), modified for printing with pre-coated pitch-based carbon fiber filaments. A customized aluminum heating block was developed to allow for dual-extrusion nozzles, extruding the pre-coated fibers from the left nozzle at a 45° angle to the bed and polymers from the right nozzle at a 90° angle, respectively. Here, the fiber-to-polymer ratio and print quality can be controlled by the arrangement of dual nozzles, as illustrated in Figure 6b. In fact, this print head is designed so that the process of mixing the polymers with the continuous carbon fiber prepreg occurs outside of the chamber. The imported prepreg can be fed through the nozzle, enabling the 3D printing of continuous carbon fiber-reinforced composites with a high-volume fraction. Obviously, the significant drawback of this method is that insufficient pressure and temperature may be provided while encapsulating the fibers and polymers outside of the print head.

## 3. Key Components of the Print Head

A print head for printing polymers typically consists of a nozzle, heating module, cooling device, chamber, guide pipe, guide gully, and other components. When working with continuous carbon fiber-reinforced composites, a cutting tool should be incorporated into the print head to assist with the cutting of printed filaments. In addition, with the introduction of continuous carbon fibers, the loss of these fibers’ mechanical properties and the interaction between the polymers and the fibers are taken into account in the structural design of components.

### 3.1. Nozzle

**Figure 7:**
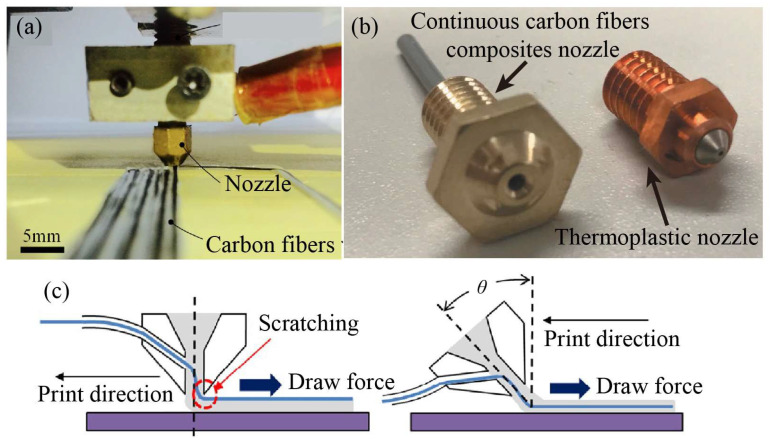
Nozzle design: (**a**) with chamfered edges [42]; (**b**) in MarkTwo printer; (**c**) with different tilt angles [47].

Nozzle clogging is the most common mechanical failure when printing continuous carbon fiber-reinforced composites. The mechanical properties and forming quality of the printed parts can be significantly influenced by the nozzles due to thermal factors and fluid–structure coupling issues. Li et al. [42] emphasized the importance of the nozzle design in preventing excessive stretching force on the filament during printing. To address this, the inside of the nozzle tip is chamfered to facilitate smooth filament extrusion and to reduce the risk of filament tearing, as shown in Figure 7a. Similarly, Markforged has developed a specialized nozzle for the MarkTwo printer’s print head, designed for continuous carbon fiber/nylon composite printing. This nozzle features a smooth edge that minimizes wear between the fiber and sharp edges [43], as seen in Figure 7b.

To withstand the abrasive nature of carbon fiber and effectively ensure proper extrusion, hardened steel, brass with wear-resistant coatings (such as nickel or chromium), and ruby-tipped nozzles are commonly used [44]. Todoroki et al. [45] observed that a conical nozzle facilitates the even mixing of continuous carbon fibers with PLA resin in a molten state. For printing carbon fiber-reinforced composites with a smooth surface, nozzles with a flattened tip are often used. Compaction force is applied during printing by adjusting the gap between the nozzle tip and the bed. Microscopic observation has validated that the reduction of fiber waviness through applying compaction force is effective [46]. 

Pappas et al. [47] investigated the effect of the nozzle tilt angle (ranging from 0° to 35°) on fiber impregnation quality to minimize the potential scratching of continuous carbon fiber, as illustrated in Figure 7c. However, the implementation of a print head design with varying tilt angles involves additional complexities to the print system. Furthermore, different nozzle diameters, materials, and print temperatures have been investigated, as shown in Table 2. 

**Table 2:** Different extrusion methods and their respective diameters, nozzle materials, and print temperatures.

Extrusion Methods	Diameter of Nozzle	Print Temperature	Material of Nozzle	References
Single extrusion	0.601 mm	80–100 °C	Brass	Zhi et al. [21]
Single extrusion	0.5–0.6 mm	200–240 °C	-	Zhang et al. [22]
Single extrusion	0.6 mm	150 °C	-	Ming et al. [23]
Single extrusion	1.5 mm	200–230 °C	Steel	Hu et al. [25]
Single extrusion	0.4 mm	–	Steel	Sugiyama et al. [26]
Single extrusion	0.9–1.0 mm	265–285 °C	Brass	Markforged printer [33]
Single extrusion	0.8–2 mm	240 °C	-	Akhoundi et al. [27]
Single extrusion	0.6 mm	–	Brass	Li et al. [42]
Single extrusion	0.6 mm	–	Ruby orifice	Olsson et al. [44]
Single extrusion	1.0 mm	–	Brass	Todoroki et al. [45]
Single extrusion	1.5 mm	260 °C	Brass	Ichihara et al. [46]
In situ co-extrusion	1.0 mm	205 °C	Brass	Kuschmitz et al. [31]
In situ co-extrusion	2.0 mm	170–180 °C	Brass	Rarani et al. [32]
In situ co-extrusion	0.8–1.0 mm	<270 °C	Steel	Anisoprint printer [35]
In situ co-extrusion	1.75 mm	180–230 °C	Brass	Yang et al. [39]
In situ co-extrusion	0.5–1.8 mm	235 °C	-	Mosleh et al. [48]
In situ co-extrusion	4.0 mm	190 °C	-	Pappas et al. [47]
Dual extrusion	1.5 mm	200–235 °C	Brass	Olcun et al. [41]

### 3.2. Heating and Cooling Block

Heating modules play a crucial role in heating and melting the polymers, allowing them to flow within the nozzle in a molten state for effective deposition and layer formation. In addition, the cooling module is instrumental in maintaining uniform heat distribution within the chamber, and this affects the temperature of the nozzle section, as demonstrated in Table 2. Note that it is essential to maintain the nozzle temperature within the normal range throughout the printing process. Zhang et al. [49] found that a temperature below the normal range may result in insufficient softening of thermoplastic composites, leading to poor bonding between adjacent fused filaments. Conversely, if the nozzle temperature exceeds the normal range, it can cause over-softening of the composites before the nozzle, resulting in adhesion issues. Heller et al. [50] employed the finite element method to numerically simulate the Stokes flow in a two-dimensional plane flow field within a polymer deposition nozzle, confirming these observations. To avoid the premature melting of the tow in the feed path, Li et al. [42] integrated a heat sink at the entrance of the guide pipe to ensure the solid state of polymer filaments. Ye et al. [51] incorporated a fan design to enhance thermal convection at the nozzle to maintain a reasonable temperature distribution.

Furthermore, the precise temperature control of the heating module plays a direct role in determining the flow viscosity of the molten resin and in influencing the curing behavior of carbon fiber filaments. Because of the remelting of impregnated filaments, there is a notable challenge in solely relying on the curing effect inside the print head to ensure the highest-quality interface between the fiber and the matrix. Due to its directly influential role in the bonding effect of the molten resin and the continuous carbon fibers, as well as the uniformity of flow in the print head, in future work, a more detailed investigation is needed in the thermal design of the print heads of continuous carbon fiber-reinforced composites.

### 3.3. Chamber

In contrast to composites manufactured by traditional molding methods, 3D-printed composites have significantly more internal defects, estimated at around 10% [31,52] and leading to lower mechanical properties. Consequently, the strength and modulus of 3D-printed composites are usually significantly lower than theoretical predictions. In the in situ immersion 3D printing process, no additional pressure is applied to enhance the penetration and diffusion of the resin. As a result, it becomes challenging to effectively remove bubbles within the fiber bundles.

**Figure 8:**
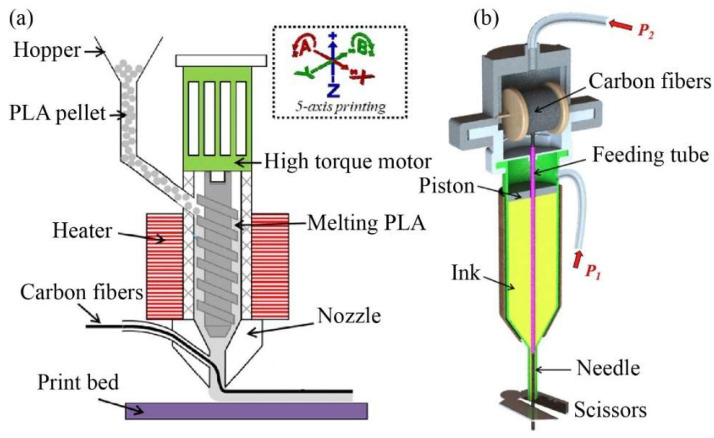
Pressure-boosting device inside the chamber of a print head: (**a**) single-screw extruder chamber [47]; (**b**) piston chamber [53].

Pappas et al. [47] used a novel single-screw extruder to ensure the deposition rate and to control the pressure inside the melting cavity. However, an increase was observed in void formation due to the decreased pressure applied to the extrudate, as shown in Figure 8a. He et al. [53] designed a print head with an embedded piston chamber that provides a deposition pressure of 0 to 100 psi through a digital pneumatic regulator, enabling high-precision pressure control in the chamber and reducing the defects inside the printed filament, as demonstrated in Figure 8b. There is no doubt that pressure control of the print head is required to expand the internal space and achieve a complex structural design within the chamber. Furthermore, the difference in pressure between the interior and exterior of the print head during the deposition process affects the occurrence of pore defects.

### 3.4. Auxiliary Parts

#### 3.4.1. Guide Pipe

The protection of the printed filament is crucial for ensuring the quality of the printed part, preventing mechanical damage and thermal issues. To address these concerns, a guide pipe was introduced to feed the printed filament into the print head [48]. Rarani et al. [32] implemented a Teflon PTFE insulation pipe to surround this metal pipe, thereby preventing direct contact between the stainless steel pipe and the filaments. These researchers proposed a numerical model to analyze the effect of thermal distribution on the print head. In addition, Hu et al. [25] introduced a PTFE tube from the inlet of the guide pipe to the end of the nozzle, which aids in ensuring consistent heating of the filament and in minimizing the friction between the pipe and the nozzle. Zhang et al. [22] found that the low viscosity of the epoxy resin in a molten state was difficult to transfer into the print head. As a solution, they implemented a PTFE guide pipe to prevent the premature melting of the printed filament of polymers. By taking these design considerations into account, the print head is able to produce high-quality prints with minimal risk of mechanical damage or thermal issues.

#### 3.4.2. Guide Pulley

**Figure 9:**
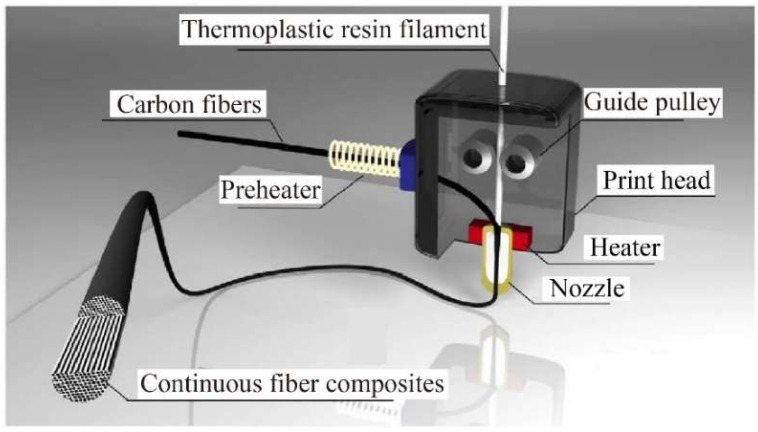
Guide pulley of print head [54].

To ensure precise control of the printing process, a guide pulley has been integrated inside or outside the print head to evenly transfer continuous carbon fiber prepregs. Matsuzaki et al. [54] utilized transmission gears and a stepper motor to convey resin filaments and continuous carbon fiber filaments in the print head, as shown in Figure 9. Additionally, the reinforcing fibers are directly delivered to the nozzle, effectively reducing the tension that may occur during the printing process and preventing damage to the printed filaments.

## 4. Conclusions

In this article, detailed descriptions of various inlet–outlet designs for print heads using fused filament fabrication have been provided. There are advantages and limitations associated with different extrusion methods:

The design simplicity of the single-extrusion print head is evident. To ensure the coordination of the fiber and matrix during extrusion, it is necessary to limit the volume fraction of carbon fibers to maintain resin fluidity. Therefore, when using a single-extrusion print head, the primary challenge is to restrict the high-volume fraction of composites. This constrains the production of high-performance composites with a single-extrusion print head.

In situ co-extruded print heads have the potential to fabricate composites with a higher-volume fraction. However, they are susceptible to poor impregnation effects, leading to local pore defects and a reduction in mechanical properties. Additionally, the impregnation process needs more pressure and time than the other two methods. This leads to lower overall printing efficiency. 

Dual-extrusion print heads provide high printing efficiency and volume fraction for continuous carbon fibers. Nevertheless, the external blending process may lead to insufficient pressure to eliminate internal pores between the fused resin and continuous carbon fibers, thereby undermining the overall quality of the molding.

It is clear that there are obvious trade-offs inherent in each extrusion method, emphasizing the imperative for sustained research to address the identified limitations and enhance the quality and efficiency of continuous carbon fiber composites. 

Indeed, the majority of manufacturing errors and equipment failures can be attributed to the structural design of the primary components of print heads. By optimizing the nozzle structure, regulating the pressure within the chamber, and ensuring the even transfer of filaments, it is possible to effectively minimize pore defects within the printing parts, ensure the desired impregnation and prevent the creasing and breakage of fibers. Furthermore, the choice of materials for nozzles and guide pipes plays a crucial role in enabling the heating and cooling modules, so as to manage temperature distribution within the printing system. This significantly influences the rheological properties of the molten resin and mitigates the risk of nozzle clogging. 

To date, structural enhancements to the components of print heads have demonstrated a significant improvement in the molding quality of continuous carbon fiber composites. However, the causes of many manufacturing issues remain unclear, including thermo-mechanical coupling, fluid-solid coupling, and rheological behavior within print heads. In light of the operational conditions within print heads, it is crucial to develop a theoretical model to analyze the effect of process parameters on the coupling mechanism. Therefore, more theoretical simulation techniques are required to support the design of the print head to improve production efficiency and the mechanical properties of continuous carbon fiber composite parts manufactured by FFF. In the future, researchers should focus on overcoming these limitations of print heads and develop theoretical models to further unlock the potential of fabricating continuous fiber-reinforced composites by FFF.

## Figures and Tables

**Table 1:** The advantages and disadvantages of the three discussed extrusion methods: single extrusion, in situ extrusion, and dual extrusion.

Extrusion Methods	Pros	Cons
Single extrusion	Simplicity, versatility, high molding quality, high degree of impregnation.	Limits the selection of constituent materials, low volume fraction of carbon fibers, low processing efficiency.
In situ co-extrusion	High volume fraction of carbon fibers, high molding quality, enhanced flexibility in the selection of constituent materials.	Complex internal structural configuration of the print head, low degree of impregnation, low processing efficiency.
Dual extrusion	High processing efficiency, enhanced flexibility in the selection of constituent materials.	(Lack of relevant reports.)

## Data Availability

The data presented in this study are available on request from the corresponding author.
